# Editorial: Plant Organ Abscission: From Models to Crops

**DOI:** 10.3389/fpls.2017.00196

**Published:** 2017-02-14

**Authors:** Timothy J. Tranbarger, Mark L. Tucker, Jeremy A. Roberts, Shimon Meir

**Affiliations:** ^1^UMR DIADE, Institut de Recherche pour le DéveloppementMontpellier, France; ^2^Soybean Genomics and Improvement Lab, Agricultural Research Service, United States Department of AgricultureBeltsville, MD, USA; ^3^Division of Plant Sciences, School of Biosciences, University of NottinghamNottingham, UK; ^4^Deptartment of Postharvest Science of Fresh Produce, Agricultural Research Organization, The Volcani CenterBet-Dagan, Israel

**Keywords:** organ abscission, abscission zone, ethylene, auxin, signaling, tomato, Arabidopsis, fruit crops

The shedding of plant organs is a highly coordinated process essential for both vegetative and reproductive development (Addicott, 1982; Sexton and Roberts, 1982; Roberts et al., 2002; Leslie et al., 2007; Roberts and Gonzalez-Carranza, 2007; Estornell et al., 2013). Research with model plants, namely floral organ abscission in Arabidopsis and leaf, flower and fruit abscission of tomato, and seed shattering in rice has provided new insights in to the molecular mechanisms underlying abscission. However, little is known about how these mechanisms that take place in the abscission zone (AZ) have diversified during plant evolution and differs between species and plant families, and within diverse tissues and developmental contexts. A major aim of this topic was to examine diverse organ abscission examples from a wide range of species in order to provide a format for comparisons between model and important crop species.

To set the stage, the Topic was spearheaded by a timely mini review on seed shattering (Dong and Wang) and a review on tomato pedicel abscission (Ito and Nakano). Dong and Wang cover recent knowledge about dry fruit dehiscence in Arabidopsis, a model dicot, and some legumes compared to seed shattering in the model monocot rice and other cereal crops and also to pedicel abscission in tomato. The review provides an overview of the importance of AZ development as a common denominator for all organ abscission to occur, pointing out commonalities and gene neo-functionalization of MADS-box genes in particular that give rise to these different dispersal systems.

Tomato flower and leaf abscission are excellent model systems to study abscission in horticultural crops: (1) tomato abscission is well characterized at the anatomical, physiological and molecular levels, especially tomato flower abscission that has a distinct flower AZ (FAZ) at the midpoint of the pedicel; (2) tomato has a high-quality, well-annotated genome available; (3) tomato plants offer opportunities for functional analyses using Virus Induced Gene Silencing (VIGS), stable transformation and recently developed gene editing methodologies; and (4) ethylene and auxin interactions in tomato abscission are well documented, which makes tomato an excellent model system for comparison to abscission in other crops discussed in this topic series (Jiang et al., 2008; Meir et al., 2010; Ma et al., 2015). Ito and Nakano provide a mini-review that describes in detail the recent advances in research on the mechanisms regulating the formation of the tomato pedicel AZ, focusing on the role of the MADS-box family transcription factors that regulate pedicel-AZ development. The review also covers recent results from transcriptome analyses that identified pedicel AZ specific transcription factors (TFs) that connect abscission-inducing signals with abscission processes.

In the article by Sundaresan et al., a *de novo* AZ transcriptome assembly provides the functional annotation of transcripts expressed in the tomato FAZ and leaf AZ (LAZ) during abscission. The results from this study revealed distinct patterns of transcriptional regulation that occur in the FAZ and LAZ, and the identification of common and distinct TFs not previously related to abscission. The AZ-specific transcript information was utilized to design a robust customized AZ microarray chip with a greater number of transcript probes than any commercial chip, including probes designed in both sense and antisense direction that can be used to explore the expression of antisense transcripts (NATs). Kim et al. used this chip to identified prominent changes in gene expression in the tomato FAZ and LAZ, in addition to parallel analysis of the soybean leaf and Arabidopsis floral AZs, linked to cell wall disassembly and synthesis, and deposition of a waxy-like boundary layer that defines the fracture plane for organ separation. They proposed that abscission is an adaptation of a more primal organogenesis process occurring in the meristem. In addition, they propose that IDA (Inflorescence Deficient in Abscission) gene expression, which appears to be a common occurrence in abscission processes, may play a role in the synthesis of the boundary layer. Tsuchiya et al. used tomato to compare the pedicel AZ cell walls during flower and fruit abscission. They provide evidence that expansin and xyloglucan endotransglucosylase/hydrolase (XTH) play important, but different roles in the FAZ during the flower abscission process, while no AZ-specific expansin and XTH expression was observed during fruit abscission, and that lignification occurred in the AZ of over-ripe tomato fruit pedicels.

The Arabidopsis floral organ abscission model was used to further explore the signaling mechanisms that control cell separation processes, with Groner et al. using suppression analysis to examine the allelic dependent restoration of organ abscission in the *nev* (*NEVERSHED*) mutant by specific alleles of *CAST AWAY* (*CST*). This work provides insight into allelic-specific interactions of key regulators of abscission, and provides a basis for nuanced applications to control abscission in crop plants. In addition, two articles focused on the Arabidopsis INFLORESCENCE DEFICIENT IN ABSCISSION (AtIDA) peptide ligand, and the leucine-rich repeat (LRR) receptor-like kinases (RLKs) HAESA (HAE) and HAESA-LIKE 2 abscission-signaling pathway (HSL2) (Jinn et al., 2000; Cho et al., 2008; Stenvik et al., 2008). In a Perspective article Estornell et al. investigated the functional conservation of the citrus IDA ortholog *CitIDA3* by over expression in Arabidopsis wild-type and *ida* mutant plants. Their results demonstrated that overexpression of the *CitIDA3* in wild-type Arabidopsis produced similar phenotypes as overexpression of *AtIDA* in wild-type Arabidopsis, which included shorter plants and siliques and early floral abscission; moreover, *CitIDA3* rescued the abscission phenotype in the *ida-2* mutant background, which provides evidence that the IDA-HAE/HSL2 abscission-signaling pathway is conserved in citrus. Furthermore, Stø et al. examined the IDA-HAE/HSL2 abscission-signaling pathway in Populus, a dicot tree, and palm (*Elaeis guineensis*), a monocot tree, and provide further evidence for conservation of function for this pathway, and that whole genome duplications gave rise to functionally divergent IDA-like (IDL) peptides during higher plant evolution. The results of these papers provide pertinent evidence that the IDA-HAE/HSL2 abscission-signaling pathway functions in higher plants in diverse phylogenetic groups. However, to what extent the IDA pathway regulates organ abscission in combination with other pathways in these species is still not clear.

Auxin and ethylene are well known to play inhibitory and positive roles, respectively, during organ abscission, and several articles further established their role during organ abscission of diverse species and organ types. Jin et al. examined the role of auxin transport during Populus leaf abscission and provided evidence that auxin transport is required to delay abscission and because the transport mechanism functions in the absence of ethylene signaling, auxin appears to act independent of ethylene on cell separation. Gao et al. performed a large-scale transcript profiling analysis during rose petal abscission, and identified the transcriptional control of important pathways in addition to 150 TFs that are differentially expressed during petal abscission in rose. In particular, down-regulation of the Aux/IAA gene *RhIAA16* in rose promoted petal abscission, suggesting that RhIAA16 plays a negative regulatory role in rose petal abscission.

The role of ethylene was examined in four manuscripts, while one manuscript examined the involvement of reactive oxygen species (ROS) during the process of fruit abscission in tropical crop species. Taesakul et al. observed different ethylene sensitivities in two distinct AZs of the tropical Longkong (*Lansium domesticum*) fruit. One AZ is more sensitive to ethylene than another adjacent AZ, the later of which is proposed to allow animals to remove ripe fruit from the tree with minimal force without a requirement for ethylene. Hagemann et al. examined the role of ethylene signaling during ethephon (an ethylene-releasing compound) induced mango fruitlet abscission. Their findings suggest a role for novel versions of the Mango ethylene receptor *MiERS1* expressed in the pedicel during premature fruit abscission. Ethylene perception in the fruitlet AZ is proposed to first reduce polar auxin transport capacity in the pedicel, followed by an up-regulation of ethylene receptors and finally a decrease in fruitlet sucrose content. Fruitlet abscission was also examined in litchi by Li et al., who performed a genome-wide digital transcript abundance analysis to identify genes regulated during ethephon-induced premature fruit abscission. The study gives insight into the major cellular processes transcriptionally regulated during ethephon-induced fruitlet abscission providing a host of candidate genes to target for control of abscission including those involved in ethylene biosynthesis and signaling, auxin transport and signaling, transcriptional regulation, protein ubiquitination, ROS response, calcium signal transduction, and cell wall modification. Yang et al. examined the involvement of ROS during the induction of longan (*Dimocarpus longan* Lour.) fruit abscission by carbon restriction caused through girdling, defoliation or detached fruit clusters. Their data suggest a role for H_2_O_2_ in the up-regulation of cellulase activity during fruit abscission, and that fruit abscission induced by carbohydrate stress is mediated by ROS. Cohen et al. examined a similar role for H_2_O_2_ in the rapid abscission of *Azolla pinnata* roots, and propose that metabolic products of nitrite and NO react with H_2_O_2_ in the apoplast that leads to free-radical-mediated cleavage of structural polysaccharides and the consequent rapid root abscission. Roongsattham et al. examined and compared the cellular processes that occur in the multi-cell layered boundary region AZ between the pedicel and mesocarp during ethylene induced ripe fruit abscission of the oil palm (*Elaeis guineensis* Jacq.). Their results highlight the dynamic developmental processes that occur in the AZ during oil palm fruit abscission, in particular changes to the pectin portion of the cell wall including a decrease in methylesterified homogalacturonan.

Flower abscission in grapevine (*Vitis vinifera* L.) induced independently by two contrasting abscission-inducing treatments: shade and gibberellic acid (GAc) was monitored (Domingos et al.). The results highlight specificities and common links in the metabolic pathways that control abscission by both inducers. While oxidative stress remediation mechanisms and a change in indolacetic acid (IAA) concentration across the AZ are common to the two abscission stimuli, GAc application and C-starvation induced abscission do not occur through the same metabolic pathways.

In the second Kim et al., a genome analysis identified 1,088 differentially regulated TFs in the soybean leaf AZ during abscission. Network analysis found that most TFs expressed early in abscission were linked to key determinants involved in the maintenance of organ polarity, lateral growth and cell fate, similar to what occurs during organogenesis in the meristem.

Hvoslef-Eide et al. compares the primary AZ in the seed funiculus of pea with the inducible secondary AZ in the poinsettia (*Euphorbia pulcherrima*) flower pedicel. The comparative analysis yielded the identification of abscission specific genes expressed in both poinsettia flower pedicel and pea seed AZs, suggesting widespread conservation of function for these gene products during organ abscission.

Lada and MacDonald provide a Hypothesis and Theory article on postharvest needle abscission in the conifer Balsam Fir. They concluded that postharvest needle abscission cannot be prevented entirely; however, abscission related processes including ethylene and other pathways identified to be involved in abscission represent targets that may delay abscission.

Finally, Patterson et al. provided a Perspective article that explores the question whether the use of model organ abscission systems such as tomato and Arabidopsis will necessarily provide applications to alter abscission in non-model understudied crop species. Their opinion highlights the challenges to translate model derived knowledge to crop species, and that it is essential for breeders and molecular biologists to work together to provide an understanding of the unique development of each species as well as the targeted genes or pathways of interest.

Overall, the current topic series of articles covered abscission of different organs in a variety of plant species including both model plants and crops. In particular, one emerging example of a common mechanism is the IDA-HAE/HSL2 signaling pathway, which is present in the AZ of a variety of species and may interact with ethylene and auxin to regulate organ abscission in these species. Articles in this series provide further evidence that this pathway functions in plants from divergent evolutionary histories, including both monocot and dicot species. Importantly, a recent article corroborates further the conservation of this pathway in the tropical fruit species litchi (Ying et al., 2016). If indeed this pathway is universally plant kingdom wide, inhibition at critical points of the pathway may allow either elimination or at least delayed organ abscission in divergent crop species. One significant development for agriculturally important but less studied crop species that is observed in this series is the next generation sequencing (NGS) based transcriptome analysis that allows rapid validation of model derived gene candidates in addition to new resources to identify targets to manage abscission in particular species and insight into the regulatory processes that may be unique to divergent species.

## Author contributions

A draft of the Editorial was first written by TT and revised by MT, JR, and SM.

### Conflict of interest statement

The authors declare that the research was conducted in the absence of any commercial or financial relationships that could be construed as a potential conflict of interest.
